# Idiopathic Retinal Vasculitis, Aneurysms and Neuroretinitis Syndrome Associated with Positive Perinuclear Antineutrophil Cytoplasmic Antibody

**Published:** 2011-10

**Authors:** Ramin Nourinia, Talieh Montahai, Nasim Amoohashemi, Hossein Hassanpour, Masoud Soheilian

**Affiliations:** Ophthalmic Research Center, Shahid Beheshti University of Medical Sciences, Tehran, Iran

**Keywords:** IRVAN Syndrome, P-ANCA

## Abstract

**Purpose:**

To report a case of idiopathic retinal vasculitis, aneurysms and neuroretinitis (IRVAN) syndrome associated with positive perinuclear antineutrophil cytoplasmic antibody (P-ANCA).

**Case Report:**

A 51-year-old man presented with loss of vision in his right eye since many years ago and blurred vision in his left eye over the past year. Ophthalmologic examination revealed optic atrophy and old vascular sheathing in the right eye and blurred disc margin, macular exudation, flame shaped hemorrhages, retinal vascular sheathing and multiple aneurysms at arterial bifurcation sites in the left eye, findings compatible with IRVAN syndrome. On systemic workup, the only notable finding was P-ANCA positivity.

**Conclusion:**

IRVAN syndrome may be a retinal component of P-ANCA associated vasculitis.

## INTRODUCTION

Idiopathic retinal vasculitis, aneurysms and neuroretinitis (IRVAN) syndrome is a rare clinical entity characterized by bilateral retinal arteritis, numerous aneurysmal dilatations of retinal and optic nerve head arterioles, neuroretinitis and uveitis. Visual loss is caused by exudative maculopathy and neovascular sequelae of retinal ischemia. This syndrome is not associated with systemic abnormalities.^1^–^3^ Antineutrophil cytoplasmic antibody (ANCA) has been described in glomerulonephritis. The presence of this antibody in the serum has emerged as a new diagnostic tool and marker of disease activity in various forms of vasculitis.^4^ Two ANCA patterns may be seen with indirect immunofluorescence studies namely, cytoplasmic (C-ANCA) and perinuclear (P-ANCA). The latter has been associated with microscopic polyarteritis nodosa (PAN) and other systemic vasculitides.^3^

Herein, we report a patient who presented with typical clinical features of IRVAN syndrome in whom systemic workup only revealed positive serum P-ANCA.

## CASE REPORT

**Figure 1:**
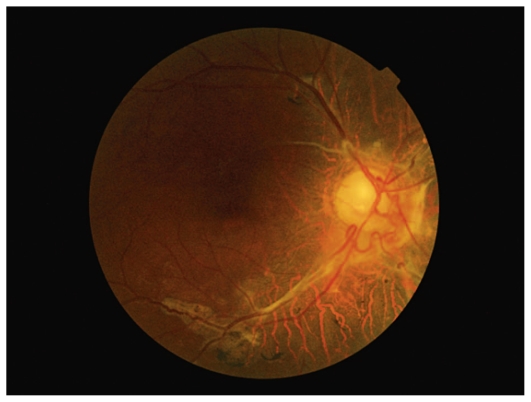
Fundus photograph of the right eye shows optic atrophy and severe old vascular sheathing.

A 51-year-old man presented with progressive blurring of vision in his left eye over the last year. Vision was no light perception in his right eye and best corrected visual acuity was 2/10 in his left eye. Slit lamp examination was unremarkable and intraocular pressure was within normal limits. Funduscopy revealed optic atrophy and severe old vascular sheathing in the right eye (Fig.1).

**Figure 2:**
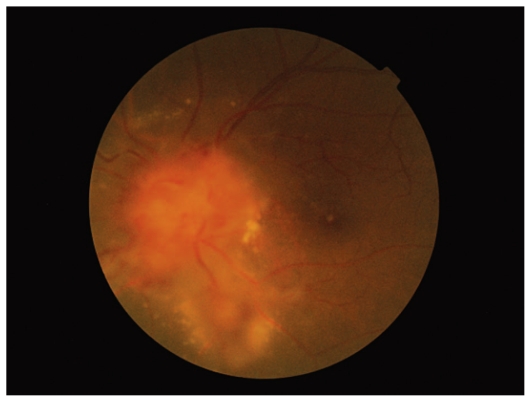
Fundus photograph of the left eye shows disc edema, hemorrhage and vascular sheathing with macular and perivascular exudation.

Posterior segment findings in his left eye included blurred disc margin with severe edema, flame shaped hemorrhages, vascular sheathing, multiple macroaneurysms at vascular bifurcation sites, together with macular and perivascular exudation (Fig.2).

**Figure 3:**
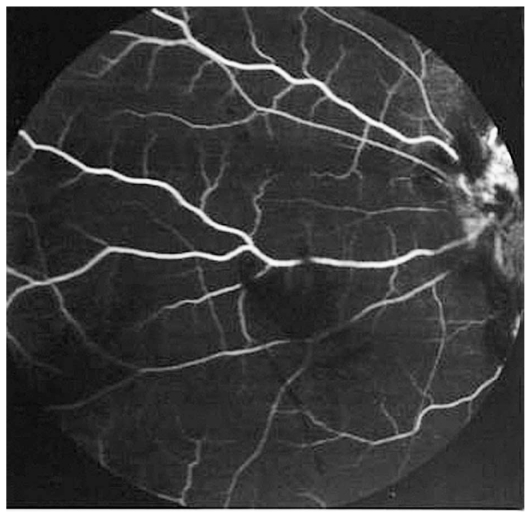
Fluorescein angiogram of the right eye reveals diffuse areas of nonperfusion.

**Figure 4:**
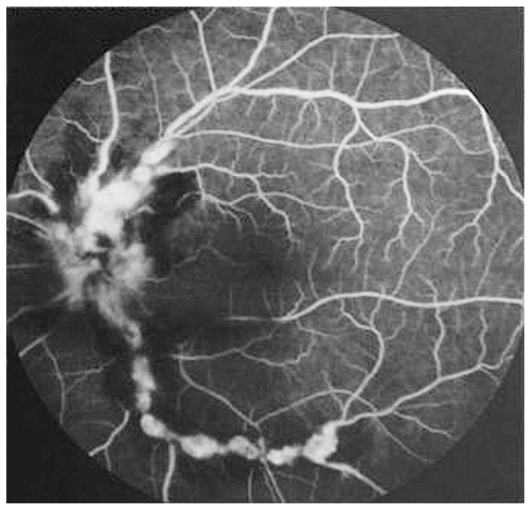
Fluorescein angiogram of the left eye reveals numerous aneurysmal dilatations at arterial bifurcation sites, focal areas of delayed choroidal filling and arterial wall staining.

Fluorescein angiography accentuated the appearance of numerous aneurysmal dilatations in retinal arterioles and areas of capillary nonperfusion (Figures 3 and 4). Systemic workup was performed and the only significant finding was positive circulating P-ANCA. Indirect immunofluorescence studies showed perinuclear fluorescence with a titer of 1:40 on 2 occasions. We consulted internists for possible systemic associations of retinal vasculitis especially Behcet’s disease, Wegener’s granulomatosis (WG), polyarteritis nodosa (PAN), tuberculosis (TB) and syphilis, all of which were ruled out.

## DISCUSSION

Kincaid and Schatz first described the association of macroaneurysms with retinal vasculitis and neuroretinitis.^5^ Samuel et al described the largest series of this condition and coined the acronym IRVAN.^2^

IRVAN syndrome is a rare form of retinal vascular inflammation predominantly affecting retinal arterioles, although choroidal vessels can also be damaged.^6^ The diagnosis is based on a constellation of clinical features. Three major criteria (retinal vasculitis, aneurysmal dilatation at arterial bifurcations and neuroretinitis) and three minor criteria (peripheral capillary nonperfusion, retinal neovascularization and macular exudation) are used to diagnose IRVAN syndrome.^1^,^2^ Three major criteria should be present for the diagnosis of IRVAN syndrome. The patient described herein had three major and two minor criteria. The etiology of this syndrome has not yet been established and it is not associated with systemic abnormalities. Differential diagnosis includes a range of inflammatory and infectious vascular diseases. Behcet’s disease, sarcoidosis, multiple sclerosis, TB, syphilis, and collagen vascular disorders such as PAN, WG and systemic lupus erythematosus may also cause neuroretinitis and macroaneurysms.^7^ Our patient demonstrated typical clinical and angiographic features of IRVAN syndrome with no association with systemic disorders; the only significant finding was positive serum P-ANCA on two different occasions during follow-up.

The prevalence of ANCA seropositivity in healthy individuals has been reported to be less than 1%.^8^ Neutrophil mediated injury of human endothelial cells is considered an important mechanism in the pathogenesis of ANCA-related vasculitis.^6^ The C-ANCA pattern has predominantly been associated with WG with sensitivities ranging from 34% to 92% and specificities between 88% and 100%,^3^ however P-ANCA is also positive in approximately 10% of WG cases. P-ANCA has predominantly been associated with microscopic PAN and is found in 50% to 80% of affected patients; nevertheless, 40% of these subjects have the C-ANCA autoantibody pattern. The average diagnostic sensitivity of P-ANCA for microscopic PAN is 15% and that of C-ANCA is 5%. These antibodies are associated with ANCA-associated vasculitis.

Since our patient was systemically normal, PAN and WG were excluded. TB and syphilis were also ruled out by normal chest X-ray and negative purified protein derivative (PPD) & venereal disease research laboratory (VDRL) tests. Based on the clinical findings and no systemic association, we diagnosed IRVAN syndrome in this otherwise healthy middle–aged man with no abnormality other than positive P-ANCA.^3^ To the best of our knowledge, only one case report has described IRVAN syndrome associated with positive P-ANCA.

The patient reported herein is a case of IRVAN syndrome with P-ANCA positivity, suggestive of a retinal form of P-ANCA associated vasculitis. We believe that, IRVAN syndrome should be added to the list of P-ANCA associated vasculitides.
